# Low reoperation rate following arthroscopic débridement using diluted povidone-iodine irrigation for septic shoulder arthritis

**DOI:** 10.1016/j.xrrt.2025.100643

**Published:** 2025-12-11

**Authors:** Terufumi Shibata, Satoshi Miyake, Kotaro Miyazaki, Kei Matsunaga, Naofumi Hata, Masahiko Sakai, So Minokawa, Yozo Shibata, Teruaki Izaki, Takuaki Yamamoto

**Affiliations:** a Department of Orthopaedic Surgery, Fukuoka University Faculty of Medicine, Fukuoka, Japan; b Department of Orthopaedic Surgery, Fukuoka University Chikushi Hospital, Fukuoka, Japan

**Keywords:** Povidone-iodine, Irrigation, Arthroscopic débridement, Septic arthritis, Shoulder, Reoperation rate

## Abstract

**Background:**

While arthroscopic irrigation and débridement are commonly used to treat septic arthritis of the shoulder because of their minimally invasive nature and favorable clinical outcomes, reinfection remains a concern. Povidone-iodine has demonstrated broad-spectrum antimicrobial activity and is increasingly used for surgical site irrigation. However, its efficacy and safety in the arthroscopic management of septic shoulder arthritis have not been well established.

**Methods:**

We retrospectively reviewed 15 shoulders in 15 patients with septic arthritis of the shoulder who underwent arthroscopic irrigation and débridement using a 0.35% povidone-iodine solution. Functional outcomes, reoperation rates for reinfection, and radiographic changes were assessed after a minimum postoperative follow-up period of 6 months.

**Results:**

Although the reoperation rate for reinfection was 0%, 1 patient (6.7%, 1 of 15) experienced reinfection, which was managed nonoperatively. No adverse effects attributable to povidone-iodine were observed. Radiographic progression of glenohumeral arthritis was noted in 4 patients (26.7%). Functional outcomes were significantly worse in patients with progressive arthritic changes compared to those without progression.

**Conclusion:**

Arthroscopic débridement combined with irrigation using diluted 0.35% povidone-iodine was associated with a low reoperation rate for reinfection in septic arthritis of the shoulder, without significant adverse effects. Further controlled studies are required to confirm the safety and efficacy of this approach.

Septic arthritis of the shoulder is a rare but serious infection of the glenohumeral joint that has a devastating impact on shoulder function, particularly if diagnosis and treatment are delayed or inadequate.^18^ Arthroscopic treatment, including débridement and irrigation, has become common due to its advantages, such as fewer complications, shorter hospital stay, and improved joint visualization, with minimal operative morbidity compared to open surgery.^15^^,^^30^ Despite improvements in surgical techniques, a recent systematic review reported that the reoperation rate after arthroscopic management remains high at 30.0%.^21^ Therefore, additional strategies may be necessary to reduce the reoperation rate due to reinfection.

Povidone-iodine is an antiseptic solution composed of polyvinylpyrrolidone, water, iodide, and 1% available iodine; free iodine is gradually released from this complex and exerts chemical toxicity against microorganisms.^8^^,^^22^ Clinically, both the World Health Organization and the Centers for Disease Control and Prevention have issued guidelines recommending intraoperative irrigation of deep or subcutaneous tissues with diluted povidone-iodine to prevent surgical site infections.^2^^,^^3^ In the management of periprosthetic joint infections, recent studies have also employed diluted povidone-iodine lavage using the same procedure.^13^^,^^16^^,^^28^ *Staphylococcus aureus*, including methicillin-resistant strains (methicillin-resistant S. aureus (MRSA)), is the most commonly identified pathogen in septic arthritis of the shoulder.^1^ Given that povidone-iodine has shown efficacy against a broad spectrum of bacteria, including MRSA and other antibiotic-resistant organisms,^4^ it is hypothesized that arthroscopic irrigation with povidone-iodine could reduce the reoperation rate due to reinfection. The efficacy and safety of diluted povidone-iodine irrigation in arthroscopic management of septic arthritis of the shoulder have not yet been elucidated. Accordingly, in this study, we aimed to describe our clinical experience with arthroscopic débridement using diluted povidone-iodine and to assess the reoperation rate due to reinfection.

## Materials and methods

This single-center retrospective study was approved by the institutional review board of our hospital. The need for informed consent was waived due to the retrospective nature of the study. We retrospectively reviewed consecutive patients aged 18 years and older who were diagnosed with septic arthritis of the shoulder and underwent arthroscopic irrigation and débridement with povidone-iodine lavage at our institution between February 2017 and October 2024. Exclusion criteria were known iodine allergy, absence of postoperative suction drains in both the glenohumeral joint and subacromial space, history of previous shoulder surgery, and postoperative follow-up of less than 6 months.

The preoperative diagnosis of septic arthritis of the shoulder was established based on the following criteria, as referenced in a previous study^29^: (1) clinical symptoms including fever, joint swelling, pain, and localized warmth; (2) laboratory findings showing elevated white blood cell count and increased C-reactive protein (CRP) levels; and (3) imaging findings on magnetic resonance imaging or contrast-enhanced computed tomography consistent with septic arthritis, including joint effusion, synovial thickening, abscess formation, and periarticular soft tissue edema. The duration of symptoms (pain, swelling, or fever in the affected shoulder) was defined as the period from the onset of shoulder pain to time of surgery. Symptom duration was recorded based on patient interviews and confirmed using clinical records or referral letters from previous hospitals. Preoperative joint fluid aspiration was performed when sufficient fluid was present in the glenohumeral joint to assess for infection. Additionally, preoperative blood cultures were obtained to identify the causative organism. The intraoperative severity of glenohumeral joint infection was assessed using Gächter's classification’ as modified by Jeon et al, which includes the following stages: stage I: turbid joint fluid, synovial membrane erythema, and possible petechial hemorrhages; stage II: marked inflammation, fibrin deposition, and purulent discharge; stage III: synovial thickening, compartmentalization, and cartilage erosion; and stage IV: invasive pannus formation infiltrating and undermining the cartilage.^15^^,^^27^ Recurrent infection was defined as the persistence of pain, swelling, fever, and limited joint mobility, with no improvement in serologic markers (white blood cell count and CRP levels) following arthroscopic intervention or a relapse of symptoms and increased serological markers after initial postprocedural improvement.^25^

### Surgical technique

**Figure 1:**
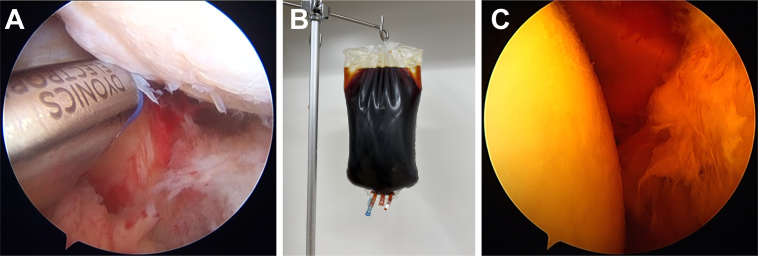
(**A**) Arthroscopic view of the left shoulder obtained through the posterior portal. A posteroinferior working portal was created at the 7 o'clock position to enable resection of synovitis involving the inferior glenohumeral capsule. (**B**) Visual appearance of a 0.35% diluted povidone-iodine solution. (**C**) Irrigation with diluted 0.35% povidone-iodine solution.

All procedures were performed by 1 of 3 experienced shoulder surgeons. After the induction of general anesthesia, patients were positioned in the beach chair position. An initial assessment of glenohumeral joint pathology was performed via a standard posterior portal. Fibrinous debris and inflamed synovial tissue were débrided using a motorized shaver inserted through the anterior working portal. Several microbiological specimens were collected for culture prior to prophylactic antibiotic administration. Joint fluid was usually aspirated from a single site within the glenohumeral joint. One or 2 synovial tissue specimens were harvested from the glenohumeral joint and/or subacromial bursa, depending on the intraoperative findings. All samples were immediately submitted for aerobic and anaerobic bacterial culture. An accessory posteroinferior working portal, positioned at the 7 o'clock orientation, was established to facilitate the resection of synovitis in the inferior glenohumeral capsule^9^ (Fig. 1, *A*). Meticulous débridement and extensive irrigation of the glenohumeral joint were performed using a motorized shaver and electrocautery. The working and viewing portals were alternated to enable débridement of the posterior capsule. The arthroscope was subsequently inserted into the subacromial space, and routine extensive irrigation and débridement were performed through the posterior, lateral, and anterior portals. When preoperative magnetic resonance imaging or contrast-enhanced computed tomography confirmed abscess formation in the extra-articular region, additional open irrigation and débridement were performed. After meticulous débridement and extensive irrigation were completed, a diluted 0.35% povidone-iodine solution was prepared by adding approximately 108 mL of povidone-iodine topical solution 10% (Meiji; Meiji Seika Pharma Co., Ltd., Tokyo, Japan) to 3,000 mL of lactated Ringer's solution (Arthromatic; Baxter International Inc., Deerfield, IL, USA) (Fig. 1, *B*). This diluted 0.35% povidone-iodine solution was administered using a pump at a pressure of 40 mmHg (Fig. 1, *C*), followed by irrigation with 3,000 mL of lactated Ringer's solution. Finally, closed suction drains were placed in all patients in the glenohumeral joint, subacromial space, and extra-articular lesion where the abscess was located. Drains were typically removed within 10 days postoperatively, depending on the volume and appearance of the drainage. All patients initially received intravenous broad-spectrum antibiotics, such as cefazolin, a first-generation cephalosporin. Antibiotic regimens were subsequently adjusted based on culture and sensitivity results and continued until CRP levels normalized. In each case, an infectious disease specialist advised on the selection, duration, and monitoring of antibiotic treatment. Patients were instructed to wear a sling until the drains were removed. After drain removal, active range-of-motion exercises were initiated. Possible adverse effects related to povidone-iodine use were clinically assessed during the postoperative follow-up period. Patients were monitored for delayed wound healing, skin irritation, or allergic reactions.

### Functional and radiological assessments

Functional evaluations were conducted at the final follow-up using the Japanese Orthopaedic Association score^14^ and the University of California at Los Angeles score.^10^ Radiographs of the affected shoulder were obtained for all patients, and osteoarthritis severity was assessed using the Samilson-Prieto classification.^26^ Postinfectious arthritis was defined by the presence of new or worsening degenerative changes in the glenohumeral joint compared to preoperative findings.^17^

### Statistical analysis

All statistical analyses were conducted using SPSS Statistics for Windows, version 30 (IBM Corp., Armonk, NY, USA). The Mann–Whitney *U* test was used to compare continuous variables between the 2 groups due to the non-normal distribution of data. A value of *P* < .05 was considered statistically significant.

## Results

**Figure 2:**
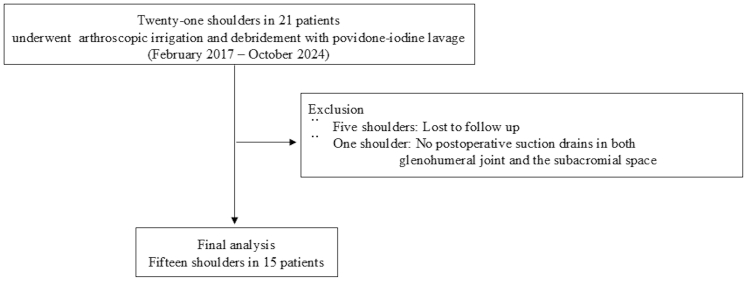
Participant flow diagram for the current study.

Among the 21 shoulders (21 patients), 15 (15 patients) met the inclusion criteria and were analyzed in the present study (Fig. 2). Detailed demographic characteristics of the patients are presented in Table I. Five patients were undergoing treatment for diabetes mellitus. The mean duration from symptom onset to surgery was 33.9 days (median, 15; range, 2-133 days). Intra-articular corticosteroid injections in the affected shoulder were suspected as the source of infection in 7 of the 15 cases (46.7%). Preoperative joint fluid aspiration and blood cultures were obtained from 14 of the 15 shoulders (93.3%).

**Table I:** Demographic characteristics of the patients.

Patient no.	Age/sex	Basic disease	Initial CRP (mg/dl)	The symptom duration before surgery (d)	Infection process	Gächter criteria	Preoperative aspiration Yes/No	Causative bacteria	JOA score	UCLA score
1	68/F	Breast cancer Diabetes mellitus	6.0	14	Injection	II	Yes	MRSA	92	32
2	70/M	Hemodialysis Diabetes mellitus	22.7	13	Injection	III	Yes	*Streptococcus agalactiae*	100	35
3	70/M	Diabetes mellitus	39.7	3	Unknown	II	Yes	*Streptococcus pneumoniae*	71.5	23
4	82/M	Diabetes mellitus	4.0	108	Injection	IV	Yes	MSSA	100	35
5	66/M	Esophagitis hypertension	1.3	9	Injection	I	Yes	*Cutibacterium acnes*	100	35
6	73/F	None	21.0	2	Injection	III	Yes	MSSA	91.5	31
7	74/M	None	4.1	133	Injection	IV	Yes	MSSA	44	8
8	78/M	Hypertension Hyperuricemia	4.0	68	Unknown	III	Yes	*Granulicatella adiacens*	82	30
9	81/F	RS3PE syndrome	40.3	3	Unknown	I	Yes	*Escherichia coli*	62	15
10	87/M	Cerebral infarction	20.6	2	Hematogenous Spread	I	Yes	*Streptococcus dysgalactiae*	94	32
11	88/M	Chronic renal failure	15.4	35	Unknown	II	Yes	*Corynebacterium striatum* MSSA	55.5	13
12	66/M	Hemodialysis	7.7	55	Unknown	I	Yes	negative	94.5	32
13	60/F	Breast cancer	7.4	18	Hematogenous Spread	III	No (dry tap)	negative	100	35
14	71/M	Atopic dermatitis Diabetes mellitus	19.5	15	Injection	II	Yes	MSSA	95	30
15	68/M	Alcoholic liver disease	2.4	30	Unknown	III	Yes	negative	95	35

*M*, Male; *F*, Female; *CRP*, C-reactive protein; *RS3PE*, Remitting seronegative symmetrical synovitis with pitting edema; *MSSA*, Methicillin-sensitive *Staphylococcus aureus*; *MRSA*, Methicillin-resistant *Staphylococcus aureus*; *JOA*, Japanese Orthopaedic Association; *UCLA*, University of California at Los Angeles.

Eleven shoulders were treated with arthroscopic débridement and irrigation alone, while an additional open procedure via the deltopectoral approach was performed in 3 shoulders with extra-articular abscess formation. In 1 case that was initially treated arthroscopically, osteolysis of the distal clavicle due to the spread of inflammation was observed, and a limited distal clavicle resection was performed through a small incision. A concomitant rotator cuff tear was observed in 11 patients (73.3%). Based on the Gächter classification as modified by Jeon, 4 shoulders (26.7%) were classified as stage I, 4 (26.7%) as stage II, 5 (33.3%) as stage III, and 2 (13.3%) as stage IV. No adverse effects were observed following the use of povidone-iodine lavage.

The causative organism was detected in 12 cases (80.0%) through analysis of either preoperative aspiration samples or intraoperative culture specimens. Intraoperative samples included joint fluid (9 of 15 shoulders) and 1 or 2 synovial tissue specimens harvested from representative areas of the glenohumeral joint and/or subacromial bursa (14 of 15 shoulders). *S. aureus* was the most frequently identified organism in intraoperative tissue cultures isolated in 6 patients. Among these, MRSA was detected in 1 case (16.7%). In 3 patients, bacterial cultures from preoperative aspiration and/or intraoperative specimens showed no growth because antibiotic therapy had already been initiated at the referring hospitals or by other departments.

The mean follow-up period was 18.0 months (range, 8-41 months). At the last follow-up, the mean Japanese Orthopaedic Association score for functional outcome was 85.5 ± 18.4 points, and the mean University of California at Los Angeles score was 28.3 ± 9.1 points. Reinfection occurred in a single case (patient no. 1), treated with arthroscopic débridement and irrigation alone, a 68-year-old woman undergoing chemotherapy following breast cancer surgery. The causative organism was MRSA. Although the initial infection after surgery for septic shoulder arthritis had resolved, reinfection developed shortly after chemotherapy was resumed 5 months postoperatively. At that time, MRSA was again isolated from the shoulder joint aspiration fluid, confirming it was the same organism identified preoperatively. Surgical intervention was recommended, but the patient declined. The infection has since been successfully managed using oral antibiotic therapy alone.

According to the Samilson-Prieto classification, 11 patients showed no signs of arthritis (normal) and 4 patients had mild or moderate arthrosis prior to surgery. At the latest follow-up, the progression of arthritic changes was observed in 4 patients (26.7%) (Table II). Among them, 1 patient (patient no. 7), whose pain and functional impairment persisted despite conservative treatment, subsequently underwent reverse shoulder arthroplasty 15 months after initial surgery. Patients exhibiting progressive arthritic changes demonstrated significantly poorer functional outcomes than those without such progression (Table III).

**Table II:** Radiographic arthritic changes based on the Samilson-Prieto classification before and after the surgery.

	Samilson-Prieto classification, n	
	Preoperatively	Last follow-up
Normal	11	9
Mild	2	2
Moderate	2	1
Severe	0	3

**Table III:** Comparison of postoperative functional outcomes between patients with and without progression of arthritic changes.

Variable	Progression of arthritic change (+) (n = 4)	Progression of arthritic change (−) (n = 11)	*P* value
JOA score (Max. 100)	64.9 ± 16.1	93.0 ± 12.9	.01
UCLA score (Max. 35)	19.0 ± 9.6	31.7 ± 6.4	.01

*JOA*, Japanese Orthopaedic Association; *UCLA*, University of California at Los Angeles.

Data are presented as mean ± standard deviation.

## Discussion

In this study, we aimed to describe our clinical experience with arthroscopic débridement using diluted povidone-iodine, as well as to assess the reoperation rate due to reinfection. Although no reoperation was required, 1 patient (6.7%, 1 of 15) developed a reinfection that was successfully managed without surgery. The reinfection occurred in a patient treated with arthroscopic débridement and irrigation alone (9.1%, 1 of 11), whereas no reinfections were observed in patients who underwent an additional open procedure (0 of 4). Consequently, the reoperation rate due to reinfection in our series was 0%. The observed reoperation rate due to reinfection appeared lower than the approximately 30% reported in a previous systematic review.^21^ Although several cases required a combined arthroscopic and limited open approach to access extra-articular abscesses, all procedures were performed according to the same principle of arthroscopic débridement and lavage. Therefore, the results can be interpreted as representing the outcomes of arthroscopic-based management for septic shoulder arthritis. Additionally, no adverse effects were observed following povidone-iodine lavage, supporting its potential safety.

Povidone-iodine has demonstrated effectiveness in various surgical fields for preventing surgical site infections^12^ and has also been utilized in the treatment of periprosthetic joint infections.^13^^,^^16^^,^^28^ Notably, unlike other antiseptics, povidone-iodine has shown no evidence of acquired resistance or cross-resistance, even after more than 150 years of clinical use.^4^ Although the optimal dilution of povidone-iodine has not been definitely established, the World Health Organization global guidelines recommend its use for irrigation, citing orthopedic spine surgery studies that employed a 0.35% diluted solution.^2^ An animal study demonstrated that povidone-iodine irrigation at concentrations of 5% or lower was safe and did not cause thyroid, kidney, or liver damage.^32^ Based on these findings, we employed a 0.35% solution, which successfully eradicated the infections. In our study, no adverse effects such as delayed wound healing, skin irritation, or allergic reactions were observed following povidone-iodine lavage, consistent with previous clinical trials.^5^, ^6^, ^7^

Kwon et al reported that technical measures such as creating an additional posterolateral portal, using a 70° arthroscope, irrigating with >20 L of normal saline, and placing multiple separate suction drains were key to reducing the reinfection rate.^20^ Similarly, in our procedure, we created an additional portal at the 7 o'clock position to remove synovitis from the inferior glenohumeral capsule and placed drains separately in the glenohumeral joint and subacromial space. Although povidone-iodine irrigation may have contributed to infection control, other technical aspects, such as extensive débridement through multiple portals and appropriate drain placement, also likely played a role in achieving a low reinfection rate.

In our cohort, only a single case of reinfection occurred, with MRSA identified as the causative organism. Takahashi et al reported that MRSA was the sole risk factor associated with the failure of initial arthroscopic intervention for shoulder septic arthritis.^29^ Povidone-iodine exhibits a broader antimicrobial spectrum—including efficacy against MRSA—than many conventional antibiotics and antiseptic solutions.^4^^,^^24^ Although the initial infection was successfully eradicated using diluted povidone-iodine, reinfection occurred shortly after the resumption of chemotherapy, 5 months postoperatively. Therefore, we believe that irrigation with diluted povidone-iodine was effective in eliminating the initial infection, even in cases involving MRSA. However, careful follow-up is necessary, particularly in immunocompromised individuals, to detect any signs of recurrence.

In this study, 4 patients (26.7%) exhibited radiographic progression of glenohumeral arthritis, and these patients demonstrated significantly poorer functional outcomes compared to those without arthritic progression. Previous studies have reported that povidone-iodine solutions exert chondrotoxic effects on the superficial layer of articular cartilage.^23^^,^^31^ Keudell et al demonstrated that povidone-iodine solutions exert chondrotoxic effects on the superficial layer of articular cartilage in a calf knee model.^31^ Among the tested concentrations (0.35%, 1.4%, 3.5%, and 5%), the 0.35% solution was the least harmful to chondrocyte viability; however, it still significantly reduced cell survival. Moreover, a strong association between chondrocyte death and osteoarthritis progression has been suggested.^19^ In this study, a 0.35% diluted povidone-iodine solution was used because it appeared less detrimental to articular cartilage. The incidence of postinfectious arthritic changes (26.7%) in our cohort was comparable to the 20%-40% reported in previous study.^17^ Based on these findings, intraoperative irrigation with 0.35% povidone–iodine appeared not to accelerate radiographic degenerative changes within the limited follow-up period of this study. However, as radiographic evaluation and follow-up duration were limited, these results should be interpreted as preliminary observations rather than definitive evidence of chondrotoxicity.

This study has several limitations. First, the minimum postoperative follow-up period of 8 months may be considered relatively short. However, the primary outcome of our study was the reoperation rate due to reinfection, and most revision procedures for infection control are typically performed within 3 days to 2 weeks after the initial surgery.^21^ In addition, infection control in septic arthritis is generally achieved within 6 months postoperatively.^20^ Therefore, we believe that a 8-month follow-up period was adequate to assess the reoperation rate for reinfection. Second, a control group was not included in our study, as we considered povidone-iodine irrigation to be an effective method for eradicating infection. Consequently, establishing a control group was not feasible. Therefore, no formal sample-size calculation was performed because this was a single-arm study. Instead, we included all consecutive cases treated during the study period and compared our outcomes with those reported in previous studies to highlight the potential superiority of povidone-iodine irrigation in achieving infection clearance. Third, because septic arthritis of the shoulder is relatively uncommon,^11^ the sample size in our study was limited. Nevertheless, the observed reoperation rate of 0% in this cohort represents a clinically meaningful improvement compared with the approximately 30% reoperation rate reported in a previous systematic review.^21^ Furthermore, the subgroup analysis comparing postoperative functional outcomes between patients with and without arthritic progression was exploratory, and the statistical power to detect a specific effect size was limited by the small sample size. Further large-scale, prospective studies are warranted to confirm these findings. Fourth, this study included a few cases in which an additional limited open procedure was combined with arthroscopic débridement to access extra-articular abscesses. Although these cases reflect real-world clinical practice,^18^ their inclusion may have introduced potential bias, as the extent of débridement and synovectomy differed from that of purely arthroscopic procedures, and the results should be interpreted with caution. Fifth, progression of arthritic changes was evaluated using plain radiographs during a relatively short follow-up period. Therefore, the assessment may not have captured subtle or long-term cartilage degeneration, and it remains difficult to determine whether these changes were attributable to the infection itself or to the intraoperative irrigation method. Future studies with longer follow-up and standardized imaging evaluation are warranted.

## Conclusion

Arthroscopic débridement combined with irrigation using diluted 0.35% povidone-iodine was associated with a low reoperation rate for reinfection in septic arthritis of the shoulder, without significant adverse effects. Further controlled studies are required to confirm the safety and efficacy of this approach.

## Acknowledgment

The authors would like to thank Editage (www.editage.jp) for English language editing.

## Disclaimers

Funding: No funding was disclosed by the authors.

Conflicts of interest: The authors, their immediate families, and any research foundation with which they are affiliated have not received any financial payments or other benefits from any commercial entity related to the subject of this article.
